# Visual analysis of the research trend and status on the association between vitamin D and immunity: From 2012 to 2021

**DOI:** 10.3389/fnut.2022.1000400

**Published:** 2022-09-21

**Authors:** Xuemei Luo, Yali Deng, Wenfang He

**Affiliations:** ^1^Department of Pediatrics, The Second Xiangya Hospital, Central South University, Changsha, China; ^2^Department of Obstetrics, The Second Xiangya Hospital, Central South University, Changsha, China; ^3^Department of Critical Care Medicine, The Second Xiangya Hospital, Central South University, Changsha, China

**Keywords:** vitamin D, immunological, hot spots, trend, visualization analysis

## Abstract

**Objective:**

We conducted this study to visualize hot spots and trends in the correlation between vitamin D and immunity over the past decade with bibliometric analysis.

**Methods:**

We collected relevant articles in the Web of Science Core Collection from 2012 to 2021 as the data source, and then used CiteSpace software to perform the data analysis. Some graphics were done with Graphpad software.

**Results:**

A total of 1,656 articles were retrieved, with an average citation count of 25.2 times. The United States (439 articles, 26.51%) has the top number of published articles, followed by China (164 articles, 9.90%), England (135 articles, 8.15%), Italy (114 articles, 6.88%), and India (82 articles, 4.95%). The most literature is found in areas of Immunology (337 articles, 20.35%) and Biochemistry Molecular Biology (179 articles, 10.81%). In terms of institutions, the top five institutions with the highest number of publications all belong to Europe. Among them, the League of European Research Universities (LERU) (121, 7.31%) has a greater proportion of output articles. The United States Department of Health Human Services (225, 13.59%) and National Institutes of Health United States (223, 13.47%) funded most articles. The leading five authors with the largest number of publications were Hewison M (19, 1.15%), Bergman P (14, 0.85%), Agerberth B (13, 0.76%), Carlberg C (12, 0.73%), and White JH (12, 0.73%). The top five keywords with the highest co-occurrence frequency are “vitamin d” (367), “d deficiency” (217), “expression” (195), “association” (151), and “d receptor” (132). Among the 17 keyword clusters, the largest cluster is #0 “diet.” Despite cluster #13 “covid-19,” most of the clusters were conducted the studies before 2012.

**Conclusion:**

The overall development of research in this field is promising. Western developed countries made outstanding contributions in this area and still take the leading role. But the participation of developing and low-income countries is also impressive. The potential therapeutic effects of vitamin D in immune-related diseases have been noted, especially in multiple sclerosis, COVID-19, etc. This is also the focus and frontier of current research. However, there is still no consensus conclusion in this field. Further research is needed in the future.

## Introduction

Vitamin D is a fat-soluble vitamin that belongs to the steroid group. Vitamin D has two forms: vitamin D2 (ergocalciferol) and vitamin D3 (cholecalciferol). Vitamin D2 is present in plants as a product of ultraviolet B (UVB) radiation from ergosterol—available as a dietary supplement or fortified in foods. Vitamin D3, the product of UVB exposure to 7-dehydrocholesterol, is either synthesized in the human epidermis or taken through dietary sources (oily fish, fortified foods, or supplements) (1). Because of the essential role of UVB in vitamin D auto-synthesis, vitamin D status is thought to be related to seasons and latitude (2, 3). Both vitamin D2 and vitamin D3 are not active and require two continuous hydroxylation steps of cytochrome P450 (CYP) enzymes to produce a fully activated form of vitamin D. Firstly, vitamin D is trafficked to the liver through vitamin D binding proteins. In the liver, vitamin D2 and vitamin D3 are hydroxylated to develop 25(OH)D by the enzyme 25-hydroxylase (CYP2R1) (4). 25(OH)D is the major circulating metabolite of vitamin D and is now the well-recognized indicator of vitamin D status due to its long half-life (5). 25(OH)D is subsequently rehydroxylated by the enzyme 1-α-hydroxylase (CYP27B1) in the kidney to produce 1,25(OH)2D, the active form of vitamin D (6). 1,25(OH)2D entails binding first to the vitamin D receptor (VDR), sequentially to the retinoid X receptor (RXR), and functions as a nuclear transcription factor, altering gene expression and inducing protein synthesis (7). Surprisingly, VDR and metabolic enzymes are expressed in a variety of immune cell types, including monocytes, macrophages, lymphocytes, and dendritic cells (8, 9). Many other tissues also express CYP27B1, including parathyroid, microglia, breast, colon, and keratinocytes, and are capable of converting 25(OH)D in circulation into the activated hormone form in an autocrine or paracrine way (10). Particularly, in such immune cells as macrophages and dendritic cells, the absence of feedback mechanisms in contrast to renal cells would instead allow the generation of high concentrations of calcitriol required for immune regulation (11).

The traditional classical role of vitamin D is to regulate calcium homeostasis and bone metabolism. However, based on these surprising discoveries that the VDR and metabolic enzyme are expressed in numerous cell types not involved in bone and mineral metabolism, the views about how vitamin D affects human health changed dramatically. Experimental studies showed that vitamin D exhibited significant biological activity in the innate and adaptive immune systems. Animal studies also suggested that vitamin D administration could result in changes in the onset and progression of diverse immune-related illnesses (12). These findings also prompted further clinical and epidemiological studies which explored the association between vitamin D and the prevalence and severity of many diseases, such as multiple sclerosis, diabetes mellitus, rheumatoid arthritis, and infectious diseases (13). Recently, Vitamin D is considered to be crucial in regulating both innate and adaptive immune functions. However, others argue that vitamin D does little for the immune system. The outbreak of the coronavirus disease 2019 (COVID-19) epidemic which ravaged the world has brought this debate to a climax. Since vitamin D deficiency is a globally prevalent health issue, the in-depth studies in this field are required and continue to be extremely valuable.

Of note, the number of research publications in this field has increased rapidly in past years. Bibliometric techniques are used for assessing and characterizing research findings and trends. Bibliometric studies allow for more objective and less biased results when comparing publication contributions from different countries, units, or individuals (14). In addition, the bibliometric analysis could be used to investigate the dynamics of a specialty, with a time-varying mapping from its knowledge foundation to its research forefront. Therefore, in recent years, bibliometrics has been widely applied in the field of medical science, and related studies are rapidly increasing (15). At present, there are few bibliometric studies on vitamin D. Most of these articles were about vitamin D on bone metabolism or COVID-19 (16, 17). Our previous study analyzed the dynamics and trends of vitamin D research in the field of infections (18). However, to date, there are no bibliometric studies that systematically describe the relationship between vitamin D and immunity. Using the bibliometric software CiteSpace, we have collected and analyzed relevant data. By presenting a realistic and intuitive overview of the development trends of research hotspots in this field, our study could provide a better insight into the research progress for scholars, physicians, policymakers, and medical students (15). We hope this study will contribute to the detection of new insights on academic trends, pharmaceutical development, and disease therapies, and support evidence-based practice in clinical education.

## Materials and methods

### Data collection

**FIGURE 1:**
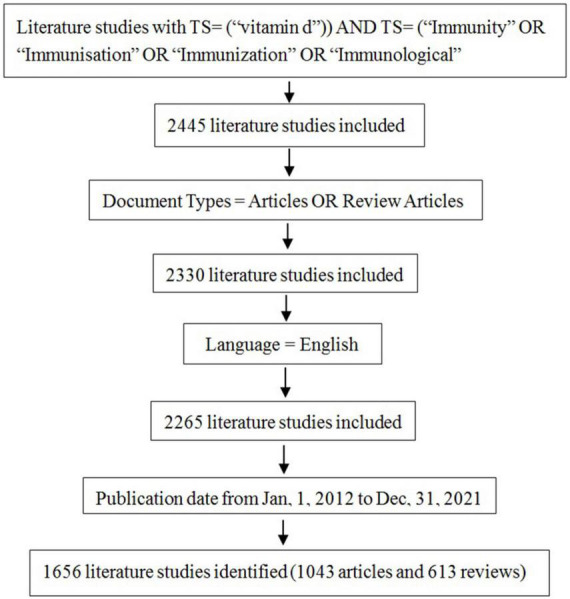
Diagram of the literature screening process. All data collection was done within 1 day on May 12, 2022. Initially, 2,445 records were retrieved from the Web of Science Core Collection (WoSCC) database. The type of articles selected for our study was limited to ARTICLE and REVIEW, and the language was limited to English. The time was set from 2012 to 2021. The final 1,656 data were obtained.

We conducted a systematic search of the literature in the Web of Science Core Collection (WoSCC) database with the following strategy: [TS = (“vitamin d”)] AND TS = (“Immunity” OR “Immunization” OR “Immunization” OR “Immunological”) AND Articles OR Review Articles (Document Types), and the search period was set from 2012 to 2021. To avoid the impact of frequent database updates, we completed all the literature searches and data collection on the date of May 11, 2022. A total of 2,445 documents were collected. The final search returned 1,656 papers, including 1,043 articles and 613 reviews. The search process was illustrated in Figure 1.

### Data analysis

The retrieved literature data were imported into CiteSpace software (version 5.8.R3) to be further analyzed (19). The detailed settings of the parameters in CiteSpace are listed below: methods (LLR), time slice (January 2012—December 2021), year/slice (1), term source (title, abstract, author keyword, and keyword plus), node type (keyword), pruning: pathfinder, selection criteria: g-index.

We evaluated the number of literature, leading countries, leading research institutions, major authors, keywords, and other indicators in the field of vitamin D and immune correlation from 2012 to 2021. Keyword co-occurrence, keyword cluster, and keyword burst graphs were drawn. The keyword co-occurrence graph consists of two parts: nodes and links. The nodes indicate the keywords and the larger the nodes, the more articles in relevant research direction. The links between the nodes refer to the connections between the keywords, and the thicker the links, the closer the associations. Keyword clusters are network groups formed by keywords with similar research topics, reflecting the evolution of themes in the field over a specified time interval. The keyword timeline cluster map brings time into the network and presents the historical trajectory and time span of the keyword evolution in each cluster. Keyword burst shows the sudden increase of keywords in a particular period, indicating a sharp growth in the popularity of a research topic in different periods. Highly cited articles were also analyzed. A comprehensive analysis of these charts could provide a more integrated picture of the development of research trends and hot spots in related fields. Microsoft Excel (version 2016) and Graphpad software (version 9.3.1) were also used for the drawings.

## Results

### General information and the global research trend

**FIGURE 2:**
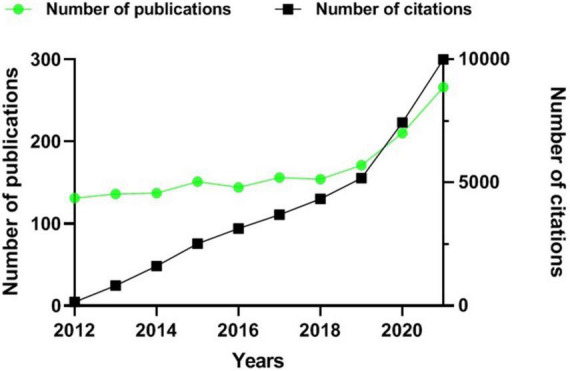
Publications and citations over time (2012–2021). The green line indicates the number of publications. The black curve indicates the number of cited articles.

The publication quantity is an essential indicator representing the trend of the research field. A total of 1,656 documents matching the limitation were retrieved. There was 30,983 literature cited, and the average citation frequency of each article was 25.2 times. As shown in Figure 2, the annual number of publications in this field gradually increased from 131 in 2012 to reach 266 in 2021. The yearly citation frequency grew from 153 in 2012 to 9,997 in 2021. These studies spanned 72 research directions, among which Immunology (337 articles, 20.35%) and Biochemistry Molecular Biology (179 articles, 10.81%) have more publications. Other favorite research areas include Nutrition Dietetics (160, 9.66%), Endocrinology Metabolism (126, 7.61%), and Pharmacology Pharmacy (110, 6.64%) (Table 1).

**TABLE 1:** Top 5 based on the publications number (2012–2021).

Field		Record count	% of 1,656
Research areas	Immunology	337	20.35
	Biochemistry Molecular Biology	179	10.81
	Nutrition Dietetics	160	9.66
	Endocrinology Metabolism	126	7.61
	Pharmacology Pharmacy	110	6.64
Countries	United States	439	26.51
	China	164	9.90
	England	135	8.15
	Italy	114	6.88
	India	82	4.95
Affiliations	League of European Research Universities	121	7.31
	University of California System	66	3.99
	Harvard University	49	2.96
	Karolinska Institutet	45	2.72
	University of London	45	2.72
Authors	Hewison M	19	1.15
	Bergman P	14	0.85
	Agerberth B	13	0.76
	Carlberg C	12	0.73
	White JH	12	0.73
Funding agencies	United States Department of Health Human Services	225	13.59
	National Institutes of Health United States	223	13.47
	European Commission	108	6.52
	NIH National Institute of Allergy Infectious Diseases	67	4.05
	National Natural Science Foundation of China	63	3.80

### Analysis of countries’ contribution

**FIGURE 3:**
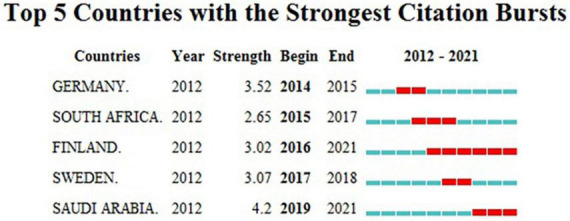
Top five countries with the strongest citation bursts (2012–2021). Burst refers to a sudden increase in the number of citations in a certain period, suggesting an increased research intensity in that country. The red bands indicate the duration of burst.

Of the 116 countries/regions involved in this research field, those with a high number of publications are the United States (439 articles, 26.51%), China (164 articles, 9.90%), England (135 articles, 8.15%), Italy (114 articles, 6.88%), and India (82 articles, 4.95%), respectively (Table 1). In addition, we carried out a further analysis of the citation burst during the last decade by countries (Figure 3). The top five countries with the strongest citation bursts are Germany (2014–2015), South Africa (2015–2017), Finland (2016–2021), Sweden (2017–2018), and Saudi Arabia (2019–2021). Among these countries, Saudi Arabia (4.2) has the highest burst strength, representing the highest research fervor in recent years. Table 1 also illustrates the top five grant funds. The United States Department of Health Human Services (225, 13.59%) and the National Institutes of Health United States (223, 13.47%) sponsor approximately the same number of articles. Other funding sources with a greater number of articles are the European Commission (108, 6.52%), NIH National Institute of Allergy Infectious Diseases (67, 4.05%), and the National Natural Science Foundation of China (63, 3.80%).

### Analysis of institutions and authors

As presented in Table 1, League of European Research Universities (LERU) has the highest number of papers (121, 7.31%) among the 2,447 participating institutions, while others include the University of California System (66, 3.99%), Harvard University (49, 2.96%), Karolinska Institutet (45, 2.72%), University of London (45, 2.72%), etc. The top five authors with the highest number of publications are Hewison M (19, 1.15%), Bergman P (14, 0.85%), Agerberth B (13, 0.76%), Carlberg C (12, 0.73%), and White JH (12, 0.73%) (Table 1). Their research subjects include the pathophysiology of vitamin d-related immunological disorders and clinical investigation. Hewison M, with an H-index of 51, works at the Institute of Metabolism and Systems Research, University of Birmingham, England. Bergman P and Agerberth B are both colleagues serving at the Karolinska Institutet, Sweden with an H-index of 32 and 55, respectively. Carlberg C is employed at the University of Eastern Finland and his H-index is 59. White J is at the McGill University, Canada with an H-index of 49.

### Analysis of research topics

#### Analysis of keyword co-occurrence and keyword cluster

**FIGURE 4:**
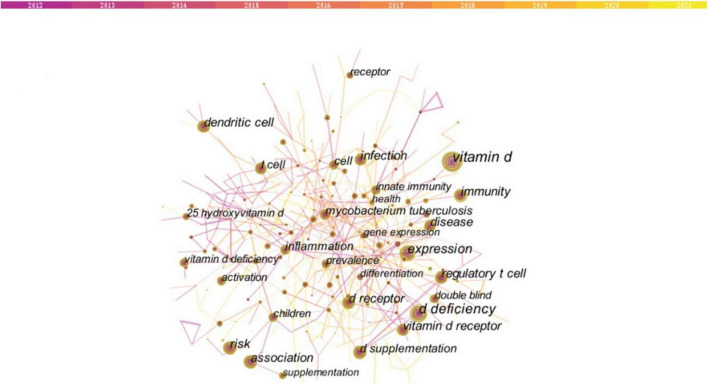
Keywords co-occurrence network (2012–2021). Nodes indicate keywords, and a larger node means that there are more research articles in that direction. The lines connecting the nodes represent the research associations between the keywords. The thicker the line, the stronger the association. The top 10 co-occurrence keywords are “vitamin d” (367), “d deficiency” (217), “expression” (195), “association” (151), “d receptor” (132), “immunity” (130), “risk” (129), “dendritic cell” (112), “disease” (110), and “regulatory t cell” (109).

Two or more keywords simultaneously presented in the same paper are regarded as one co-occurrence. Keyword co-occurrence graph is predicated on the frequency of keyword co-occurrences in the cited literature. Keyword co-occurrence analysis facilitates the identification of research hotspots and trends in the field. As shown in Figure 4 and Table 2, the keywords with high co-occurrence frequency include “vitamin d” (367), “d deficiency” (217), “expression” (195), “association” (151), “d receptor” (132), “immunity” (130), “risk” (129), “dendritic cell” (112), “disease” (110), and “regulatory t cell” (109).

**TABLE 2:** Keywords co-occurrence frequency (Top 35 in count order, 2012–2021).

Keywords	Count	Centrality	First appearance year
Vitamin d	367	0	2012
D deficiency	217	0	2012
Expression	195	0	2012
Association	151	0.01	2012
D receptor	132	0.08	2012
Immunity	130	0.01	2012
Risk	129	0	2012
Dendritic cell	112	0.03	2012
Disease	110	0.02	2012
Regulatory t cell	109	0.02	2012
Vitamin d receptor	108	0	2012
Infection	106	0.01	2012
D supplementation	103	0.09	2012
T cell	93	0.04	2012
Cell	91	0.13	2012
Mycobacterium tuberculosis	86	0.03	2012
Inflammation	85	0	2012
Double blind	78	0.02	2012
Activation	77	0	2012
Innate immunity	76	0.01	2012
Vitamin d deficiency	74	0.02	2012
Children	68	0.11	2012
Prevalence	67	0.02	2012
Receptor	61	0.01	2012
25 hydroxyvitamin d	61	0.02	2012
Health	57	0.15	2012
Supplementation	55	0	2013
Differentiation	53	0.06	2012
Gene expression	52	0.03	2012
Prevention	49	0.1	2012
Crohns disease	46	0.17	2012
Immune system	46	0	2012
*In vitro*	45	0.15	2012
Multiple sclerosis	45	0.01	2012
1 alpha	45	0.07	2012

**FIGURE 5:**
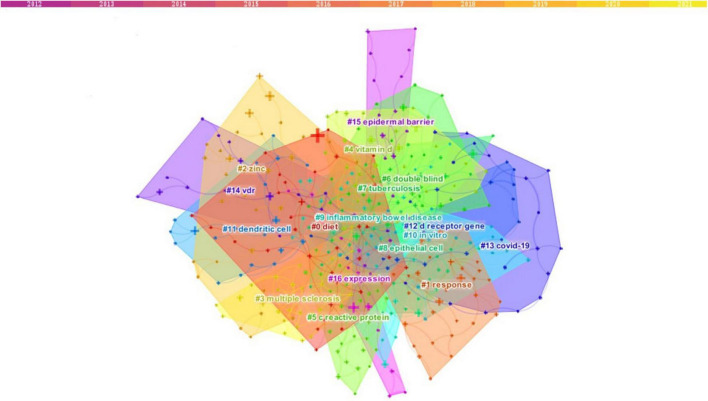
Keyword cluster analysis (2012–2021). There are 17 clusters in all, distinguished by different colors. Cluster #0 is the largest, following by cluster #1, and so on. The topic term used more frequently in relevant articles was assigned by CiteSpace as the cluster label. The top five clusters are “diet,” “response,” “zinc,” “multiple sclerosis,” and “vitamin d”.

Based on the keyword co-occurrence map, it can be found that the keyword co-occurrence network has gathered into an irregular area, namely a cluster. Briefly, the keyword cluster is a network consisting of keywords with similar study topics to discover the main subjects. In total, 17 separate clusters were obtained. Within each cluster, the topic term used more frequently in articles serves as the cluster marker by CiteSpace. In the calculation rules of CiteSpace, clusters are sorted from zero. That is, cluster #0 is the largest cluster; cluster #1 is the next largest cluster, and so forth. According to the keyword cluster analysis, the five largest clusters are “diet,” “response,” “zinc,” “multiple sclerosis,” and “vitamin d” (Figure 5 and Table 3). In cluster #0, the associated keywords include “obesity” and “nutrition.” In cluster#1 “response,” the most appeared keywords are “d receptor,” “mycobacterium tuberculosis,” “experimental autoimmune encephalomyelitis” and “asthma.” In cluster #2 “zinc,” the keywords mentioned mainly include “supplementation,” and “adolescent.” In cluster#13 “COVID-19,” the most prevalent keywords are “ace2,” “severe acute respiratory syndrome coronavirus 2,” “renin-angiotensin-aldosterone system” and “sars-cov-2.”

**TABLE 3:** Cluster summary (2012–2021).

ClusterID	Label (LLR)	Size	Mean (year)
0	Diet	35	2015
1	Response	33	2014
2	Zinc	30	2014
3	Multiple sclerosis	29	2013
4	Vitamin d	28	2015
5	C reactive protein	27	2015
6	Double blind	27	2015
7	Tuberculosis	27	2014
8	Epithelial cell	26	2015
9	Inflammatory bowel disease	26	2014
10	*In vitro*	23	2015
11	Dendritic cell	19	2013
12	D receptor gene	18	2016
13	Covid-19	17	2020
14	Vdr	17	2016
15	Epidermal barrier	17	2016
16	Expression	13	2014

It could be concluded that the association between vitamin D and immune function received long-standing attention during the past decade, especially in immune-related diseases such as multiple sclerosis (MS), tuberculosis (TB), experimental autoimmune encephalomyelitis, and asthma. COVID-19 is also a hot concern.

#### Analysis of keyword cluster timeline and keyword bursts

**FIGURE 6:**
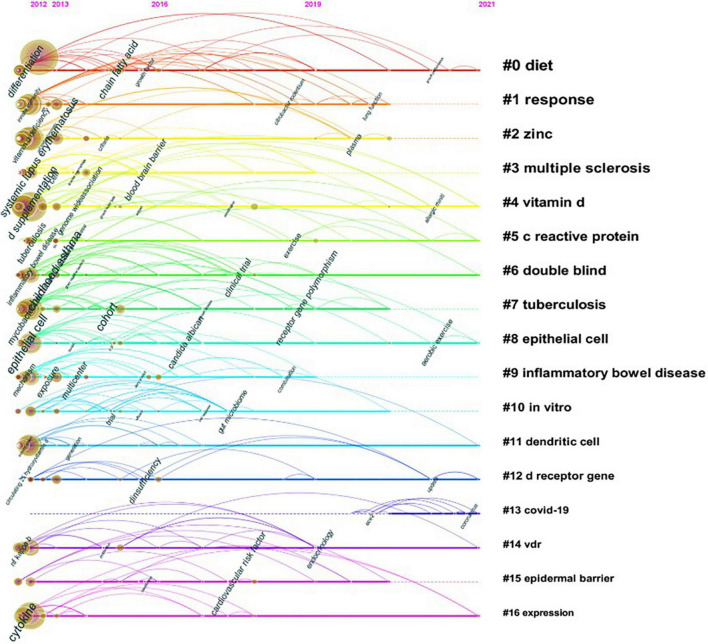
Timeline view of keyword cluster. Timeline maps are used to show the evolution of research hotspots in a time-based manner. The length of the horizontal straight line of a cluster indicates its time frame.

The keyword timeline graph after clustering was plotted using Timeline view in CiteSpace (Figure 6). In the timeline view, these keywords are distributed in their corresponding clusters according to the year in which they appeared. The length of the horizontal line of each cluster indicates its timeframe. The timeline view reveals the historical span of the literature visually and is used to track the progression of research trends. As presented in Figure 6, the study of cluster #13 “COVID-19” started in 2019, coinciding with the sudden outbreak of the COVID-19 epidemic. Apart from that, most of the cluster studies started before 2012.

**FIGURE 7:**
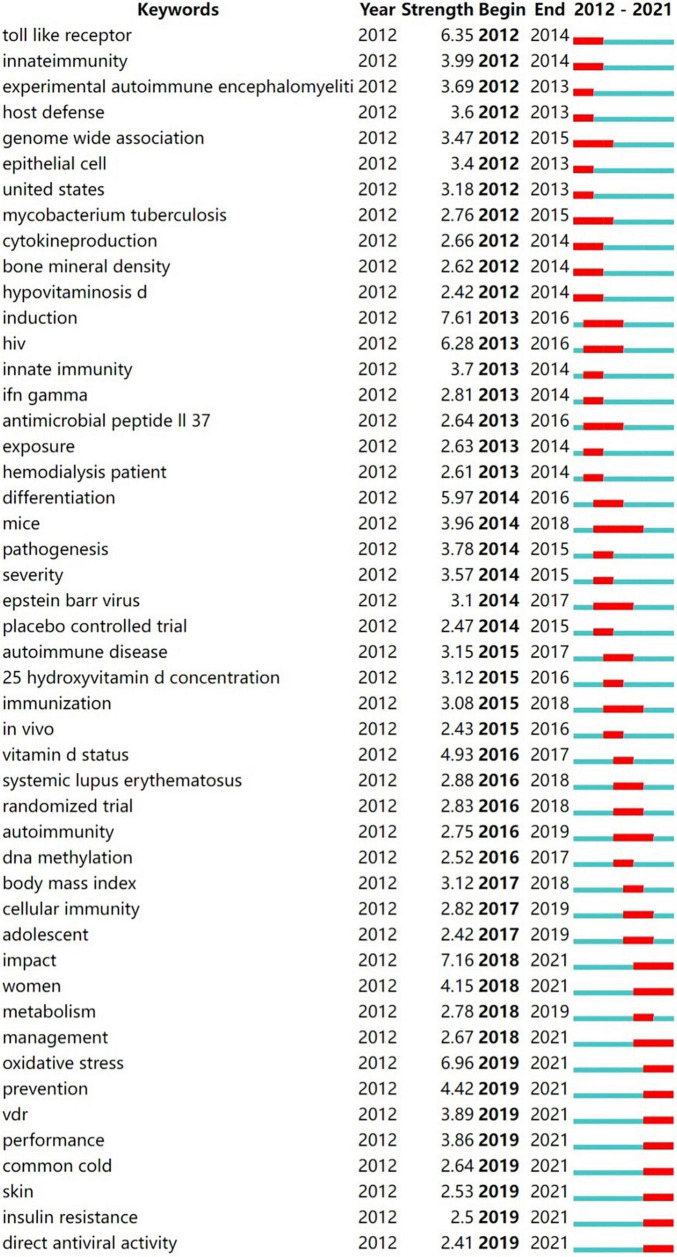
Keywords with the strongest citation burst (2012–2021). The red line shows the duration of the keyword citation burst, indicating the progress of the frontier hot topics.

We further mapped the keyword citation burst with CiteSpace (Figure 7). In general, the keyword burst is a sudden increase of keywords citation in a specific research area at a specific time. The red line shows the citation burst duration, indicating the progression of hot topics. As illustrated in Figure 7, a total of 48 keywords burst emerged from 2012 to 2021, with “induction” (7.61) having the highest intensity. The keywords burst lasting more than 4 years include “genome wide association” (2012–2015), “mycobacterium tuberculosis” (2012–2015), “induction” (2013–2016), “hiv” (2013–2016), “antimicrobial peptide II 37” (2013–2016), “mice” (2014–2018), “epstein barr virus” (2014–2017), “immunization” (2015–2018), “induction” (2013–2016), “autoimmunity” (2016–2019), “impact” (2018–2021), and “women” (2018–2021). The result indicated that research in these directions have received a high level of attention from researchers and have a long duration of popularity. The latest burst keywords include “oxidative stress” (2019–2021), “prevention” (2019–2021), “vdr” (2019–2021), and “insulin resistance” (2019–2021), and so on.

It can be summarized that the research contents on vitamin D in immunity in the past decade include specific molecular mechanisms, studies with animal models, and clinical trials, especially in the applications in immune-related disorders.

#### Analysis of highly cited articles

Highly cited articles can reflect the hot spots of the research. Table 4 shows the top ten most cited articles on the association between vitamin D and immunity. Three of these ten articles (ranked 4th, 9th, and 10th) discussed the relationship between vitamin D and multiple sclerosis. The third- and sixth-ranked articles overviewed the role of vitamin D in the human immune system and the possible mechanisms. The second- and third-ranked articles also addressed the link between vitamin D and multiple sclerosis and other immune-related conditions. The fifth- and eighth-ranked articles focus on the role of vitamin D in anti-inflammatory and anti-infection. These heavily cited articles showed the consistent interest in the association between vitamin D and immunity over the past decade. In addition, the association of vitamin D in multiple sclerosis has certainly been the focus of attention.

**TABLE 4:** Top 10 high-cited articles related to vitamin D and immunology.

Ranking	Title	Authors	Journal	Year	Citations
1	The IOC consensus statement: beyond the Female Athlete Triad-Relative Energy Deficiency in Sport (RED- S)	Mountjoy, M; Sundgot-Borgen, J; Burke, L et al.	British Journal of Sports Medicine	2014	565
2	Vitamin D effects on musculoskeletal health, immunity, autoimmunity, cardiovascular disease, cancer, fertility, pregnancy, dementia and mortality-A review of recent evidence	Pludowski, P; Holick, MF.; Pilz, S et al.	Autoimmunity Reviews	2013	511
3	Vitamin D and Immune Function	Prietl, B; Treiber, G; Pieber, TR. et al.	Nutrients	2013	474
4	Interactions between genetic, lifestyle and environmental risk factors for multiple sclerosis	Olsson, T; Barcellos, LF.; Alfredsson, L	Nature Reviews Neurology	2017	407
5	A comprehensive summary of LL-37, the factotum human cathelicidin peptide	Vandamme, D; Landuyt, B; Luyten, W et al.	Cellular Immunology	2012	361
6	An update on vitamin D and human immunity	Hewison, M	Clinical Endocrinology	2012	333
7	So depression is an inflammatory disease, but where does the inflammation come from?	Berk, M; Williams, LJ.; Jacka, FN.; et al.	BMC Medicine	2013	323
8	Why does COVID-19 disproportionately affect older people?	Mueller, AL.; McNamara, MS.; Sinclair, DA.	Aging-Us	2020	293
9	Role of the innate and adaptive immune responses in the course of multiple sclerosis	Hemmer, B; Kerschensteiner, M; Korn, T	Lancet Neurology	2015	291
10	Clinically isolated syndromes	Miller, DH.; Chard, DT.; Ciccarelli, O	Lancet Neurology	2012	284

## Discussion

### General tendency of research

As seen in Figure 2, the growth of articles on vitamin D and immunity was relatively stable in each year before 2019. After 2019, the significant increase in the number of articles in this research area should be related to the outbreak of COVID-19. About twice as many issues in 2021 as in 2012. Interestingly, there has been notable growth in the number of citations over the past decade, from hundreds in 2012 to nearly ten thousand in 2021 (Figure 2). This also indirectly reflected the overall favorable development of research in this field. Our results are consistent with previous studies (17, 20). Their findings suggested that in recent years, the research hotspot for vitamin D has changed significantly from bone metabolism to other fields, such as neuroscience, cardiovascular diseases, cancer, as well as immune-related diseases.

As presented in Table 1, the United States, China, England, Italy, and India are the top five countries with the highest number of publications. Notably, the number of articles contributed by United States scholars is almost the same as the total number of publications from the other following four countries together. Among the top five institutions—the LERU, the University of California System, Harvard University, Karolinska Institutet, and the University of London—two belong to the United States and three to Europe. Of the five grants with the highest number of sponsored articles, three come from the United States, one from Europe, and the other from China (Table 1). With ample financial support, the strong academic atmosphere, and leading research institutions, it is understandable that the United States ranks top in the number of publications. Of the five most prolific authors, Bergman P and Agerberth B are both from Karolinska Institutet. Moreover, these two authors account for more than half of the total number of publications by the affiliated institution. It is worth noting that although China ranks second in terms of the number of publications, neither the top five research institutions nor the top five authors are from China. Similarly, among the top 10 highly cited articles, no paper was written by Chinese scholars (Table 4). These data indicate that the influence of Chinese academics and research institutions in this field is relatively limited compared to that of developed Western countries. The lack of impact of Chinese scholars may be related to the weak international collaboration (17). The same situation is present for India. Developing countries need further in-depth research in the future to enhance their impact. As shown in the Figure 3, Saudi Arabia, a developing country, has also shown a high level of enthusiasm for research and has conducted active studies in recent years. It should be aware that although vitamin D deficiency is considered a global issue, it is worse in less developed countries. According to a global study on the prevalence and disease burden of vitamin D deficiency, India (61%), Iran (86%), and Turkey (51%) were associated with high rates of severe vitamin D deficiency in infants, while the incidence of vitamin D deficiency in these countries was 90% or worse (21). In addition, despite abundant sunlight, vitamin D deficiency is common in Middle Eastern countries (22, 23). A fully covered clothing style, reduced outdoor activity due to the hot summer months, and dietary customs may contribute to the poor vitamin D level in these countries (24, 25). We should be pleased with the growth of studies being conducted in these low-income and less developed countries. It is also expected that these studies on vitamin D will draw the awareness of the authorities in these countries and enable them to initiate necessary actions.

In general, our results show the alignment of highly productive authors, leading institutions and leading countries, and funding agencies. Furthermore, our study indicates the pioneering position of Western developed countries in the field. Developing countries and low-income countries have also made significant contributions to this area.

### Evolutions and focuses of research

The shift in research hotspots was illustrated in Figure 7. The focus of the research was initially on the role of vitamin D in the pathophysiological process of disease. Thus, the popular keywords included “toll like receptor” (2012–2014), “host defense” (2012–2013), and “cytokine production” (2012–2014). As the knowledge of the functioning mechanism of vitamin D gradually improved, the research hotspots accordingly transitioned to the treatment and even prevention of vitamin D for immune-related diseases. Hence, “mice” (2014–2018), “randomized trials” (2016–2018), “management” (2018–2021), and “prevention” (2019–2021) have emerged as research hotspots recently. Of note, as the Timeline View illustrates (Figure 6), the developments of these research hotspots are not isolated; they are intertwined and progressing together. Since the detailed molecular mechanisms of vitamin D in immunity have not been elucidated, this crossover development is rational. Vitamin D is currently being extensively studied in many immunological diseases, especially MS and TB, as shown in our results (Figure 4 and Tables 3, 4).

Multiple sclerosis is an inflammatory neurodegenerative demyelinating disease of the central nervous system (CNS), most probably of autoimmune origins. The fourth most highly cited article (Table 4) reviewed the literature and indicated that low vitamin D was an underlying risk factor in the development of MS (26). The third-ranked article (Table 4) also reviewed the impacts of vitamin D on the innate and adaptive immune system, with a special focus on its research progress in MS, such as possible molecular mechanisms, therapeutic effects, etc. (10). These highly cited articles have also contributed to the flourishing research on vitamin D in MS. Meanwhile, MS lacks effective therapeutic drugs and patients suffer from poor life quality, while vitamin D supplementation is both inexpensive and easy to monitor. It is therefore not surprising that a large number of studies have focused on the relationship between vitamin D deficiency and the incidence of MS. The extensive epidemiological studies supported the causal relationship between low vitamin D levels and MS onset and progression (27). In a previous groundbreaking study, Munger and colleagues demonstrated that increasing vitamin D concentrations, especially before the age of 20 years, was related to decreasing the later risk of developing MS (28). Further study revealed that vitamin D supplementation at the ages of 13–18 could reduce the risk of MS (29). These findings were consistent with the results from the experimental autoimmune encephalomyelitis (EAE) animal model studies. A study in the EAE model of MS indicated that vitamin D levels only affected the incidence and course of disease in adolescent rats, but not in pregnant or adult ones (30). These findings reinforced that adolescence was a critical period of susceptibility for developing MS in adults. Therefore, it is not surprising that the keyword “adolescent” is a hot research topic (Figure 6).

Another bibliometric study on vitamin D in neurodegenerative diseases also showed that vitamin D was most studied in MS, especially for its use as a biomarker, while the genetic aspects of this molecule were less studied (31). However, our results suggested the popularity of the keyword “genome wide association” (Figure 7). Genome-wide association studies (GWAS) have already identified that genetic abnormalities in genes implicated in vitamin D metabolism, such as specific *CYP27B1*, and *CYP24A1*, are associated with an elevated risk of MS (32). A Mendelian randomization study with data from the SUNLIGHT Study, the largest GWAS of vitamin D so far, identified four single nucleotide polymorphisms (SNPs) (rs10741657, rs12785878, rs2282679, rs6013897) that were related to lower vitamin D concentrations and increased susceptibility to MS (33). This study, together with the other three independent Mendelian randomized studies consistently concluded that individuals with genetically motivated lower serum 25(OH)D levels were at elevated risk of having MS either in adolescence or adulthood (34). The latest Mendelian randomization study assessed data from the International Multiple Sclerosis Genetics Consortium discovery phase GWAS, using six SNPs linked to serum 25(OH)D levels and found them to be associated with an increased risk of MS onset (35).

Many studies further posed the issue of the possibility of vitamin D supplementation as a therapeutic agent for MS. Thus it is reasonable that keywords including “randomized trials” (2016–2018), “management” (2018–2021), and “prevention” (2019–2021) become popular (Figure 7). In an observational study, high vitamin D levels were associated with reduced axonal damage in MS patients as evaluated by the cerebrospinal fluid neurofilament light chain level, a sensitive biomarker of neuronal axonal injury (36). Similar results were obtained in another large randomized controlled trial (RCT) containing 1,482 participants. It showed that in MS patients treated with interferon beta-1b, higher 25(OH)D levels were correlated with a lower rate of MS activity measured on MRI (37). However, different studies yielded different results. Recently, several clinical RCTs revealed that high-dose vitamin D_3_ supplementation for 48 or 96 consecutive weeks failed to present benefits on neurofilament light chain levels (38, 39). Another RCT study recruited 229 relapsing-remitting MS (RRMS) patients and treated them with subcutaneous interferon β-1a therapy. These individuals were also randomized to add the placebo or vitamin D_3_. The effectiveness of adding vitamin D_3_ was then evaluated. Interestingly, although 48 weeks of vitamin D supplements did not improve the endpoint of disease activity status, the results still suggested a protection against the progression of fresh MRI lesions in RRMS patients (40). This finding was in line with a previous study in which vitamin D_3_ supplementation alongside interferon β-1b treatment reduced MRI disease activity in MS (41). In general, there is still a lack of conclusive evidence for the benefit of vitamin D supplementation in MS. The dispute in this research area continues, thus it is natural that the topic stays popular.

Our results also suggested that VDR received long-standing attention in immune-related diseases (Figures 6, 7), which was consistent with another bibliometric study on VDR (42). As addressed in the previous section, VDR mediates much known physiological functions of the active form of vitamin D, 1,25(OH)_2_D. Upon binding to 1,25(OH)_2_D, VDR is heterodimerized with the retinoid X receptor in the nucleus, which then combines with the vitamin D-response element (VDRE) in the promoter of the target gene, thereby initiating gene transcription (7). In fact, approximately 3% of the mouse and human genomes are directly or indirectly regulated by vitamin D, suggesting a broad contribution of vitamin D/VDR in diverse disease mechanisms (43, 44). For several organ dysfunctions, the vitamin D/VDR signaling pathway is essential in regulating connectivity components and maintaining epithelial barrier integrity, including intestinal inflammation, infection, and chronic inflammatory lung diseases (45). It was previously proposed that the main mechanism by which VDR activation affected autoimmunity was through genetic activation of myeloid immune cells, particularly antigen-presenting dendritic cells (DCs), thereby triggering a tolerance status in the immune system, particularly in DCs (46, 47). More recently, it has been shown that VDR bound to thousands of loci in the genomes of human monocytes and DCs, about half of which possessed typical VDRE motifs (48). This study further suggested that the interference with VDR binding at certain relevant disease risk gene variants may facilitate the susceptibility to latitude-dependent autoimmune diseases, such as MS. Recently, the promising role of VDR as a therapeutic target has been acknowledged (49). Thus, it is understandable that keywords including “vdr,” “d receptor gene,” and “dendritic cell” are attracting more attention (Figures 6, 7). However, to date, there is no safe and effective method of modifying VDR activity. Additional research is required in the future.

Besides MS, our results showed the interest of researchers in the role of vitamin D in tuberculosis (Figure 6 and Table 2). The fifth most highly cited article reviewed related insights on human cathelicidin LL-37 in immune regulation and response (50) (Table 4). This article also highlighted the role of vitamin D and VDR in the regulatory mechanism of cathelicidin peptide expression. It was revealed that vitamin D mediated the host response to Mycobacterium infection through the induction of the antibacterial peptide cathelicidin (51, 52). Therefore, various RCTs of vitamin D supplementation for the treatment and prevention of TB were conducted. Interestingly, vitamin D was used to treat TB long before the availability of antibiotics. Niels Finsen was awarded the 1903 Nobel Prize in Physiology or Medicine for his success in treating disease, particularly cutaneous tuberculosis, with exposure to the arc lamplight. However, those RCTs yielded mixed results. One RCT study conducted by Martineau and colleagues showed that 2.5 mg vitamin D_3_ supplementation had no significant effect on sputum culture conversion time in the entire study population, but had a significant accelerated effect on sputum culture conversion in participants with the *Taq*I VDR polymorphism (53). In another RCT in Mongolia enrolling 390 adult TB patients, supplemental vitamin D_3_ only speeded sputum culture conversion in individuals carrying one or more SNP minor alleles in the gene encoding the *VDR* and *CYP27B1*, while remaining unaffected in the overall population (54). The most recent large RCT study that recruited 4,000 adults living with HIV suggested that vitamin D supplementation did not reduce overall mortality or TB incidence when compared to the placebo group (55). However, a recent RCT study in Indonesian TB children showed that vitamin D supplementation could improve fever and cough (56). Given that TB is the 13th cause of death worldwide and the second leading cause of infectious disease following COVID-19 (57), it warrants continued research in this area.

### Research frontiers and future prospects

As mentioned in the previous section, the role of vitamin D in immune regulation and related diseases has been widely discussed, yet it remains inconclusive. The outbreak of the COVID-19 epidemic further fuels the debate. Hence, it is not surprising that related studies are at the frontier of research (Figure 6). Extensive studies confirmed the association between low vitamin D levels and COVID-19, including disease severity, incidence, and mortality (58–60). The ninth-ranked high-cited article addressed the issue of vulnerability in older adults (Table 4) and suggested low vitamin D status as a possible factor (61). However, some studies concluded that no causal link was identified between vitamin D deficiency and COVID-19 (62, 63). The debate on the relevance of the two continues, while research on vitamin D in disease treatment and prevention has been conducted. However, different RCTs came to different conclusions either (64, 65). To date, three bibliometric studies on vitamin D and COVID-19 are available. Two of them used Elsevier’s Scopus database, which was different from the WoSCC database of our studies (66, 67). The remaining other study chose to use the WoS database and concluded that the direction of research on vitamin D and COVID-19 changed over time. According to their results, recent research trends focused on vitamin D deficiency and the incidence of disease, and the promise of vitamin D supplementation in treating COVID-19 (16). Interesting, our findings showed that the most popular keywords in cluster#“COVID-19” included “ace2” and “renin-angiotensin-aldosterone system,” suggesting that the associated molecular mechanisms were also of interest. The difference in these results is related to the fact that our study focused on vitamin D and immunity, while their study emphasized more on the association of vitamin D with COVID-19. Xu and colleagues indicated that vitamin D could protect rats from lipopolysaccharide-induced acute lung injury by modulating the angiotensin-converting enzyme 2 (ACE2) expression *via* the renin-angiotensin system (RAS) (68). It was further illustrated that following COVID-19 infections, downregulation of ACE2 and impairment of RAS may be responsible for multi-organ damage (69). Currently, outbreaks due to mutated strains of the virus are still prevalent worldwide. Research in this area remains urgent.

Many factors contribute to the varied findings of these studies about vitamin D. It should be noted that these trials differed regarding the region, ethnicity, amount, age, and baseline vitamin D levels of the recruited population. The supplementation regimens also varied. In particular, the RCT design protocol for vitamin D is quite different from those for generic drugs. One essential factor is that any conclusions about the beneficial effects of vitamin D supplementation on health must be validated by baseline and post-treatment vitamin D status in the study population (70). Researchers should pay attention to rational designs when conducting future investigations. In addition, vitamin D status is inherently influenced by numerous factors, such as vitamin D binding proteins, genetic factors, seasons, latitude, lifestyle, etc. (71, 72). Although 25(OH)D is recognized to be the most reliable indicator of vitamin D status (73), there is still controversy regarding vitamin deficiency and insufficiency, as well as the appropriate dose of supplementation. Overall, despite several advances, our understanding of this field is still lagging. The detailed mechanisms by which vitamin D is engaged in the immune system have not been entirely elucidated. Moreover, low vitamin D levels are relatively prevalent among individuals of all ages worldwide (72). While the way to improve vitamin D status is simple and inexpensive, relevant authorities should be concerned about this issue and take appropriate measures. Even a modest experimental benefit could have a significant public health impact on a large population. It is expected more evidence of vitamin D benefits will emerge in this field.

### Limitations

Compared with conventional literature reviews, the bibliometric analysis of CiteSpace provides much better insight into the research trends and hotspots, as well as a more comprehensive and objective view of data. Our study presents the first bibliometric analysis of vitamin D concerning immune function, but it still has several limitations. Firstly, we performed the literature search only in the WoSCC database. While it ensures the accuracy of data, such as author names, it may overlook some articles that are only available in other databases. Meanwhile, since the database is continuously updated, the results of this study are somewhat different from the actual number of available literature. In addition, only articles and reviews were selected for our study, but the papers were of mixed quality. Last but not least, only papers in English were collected in this study and we may miss some articles published in other languages. However, we still believe that our visual analysis could give scholars a quick overview of the whole research situation and frontiers in this area.

## Conclusion

While Vitamin D’s role is widely recognized in bone health, its capability to modulate immune responses has been extensively studied over the past decade. Our study systematically evaluated the role of vitamin D in this area, using a bibliometric analysis of 1,656 publications collected from the WoSCC database from 2012 to 2021. Our findings highlight the significant influence and contribution of developed Western countries, represented by the United States, in this area. It is also noted that many developing countries and low-income countries/regions are engaged in research on this topic. The potential contribution of vitamin D to the treatment and prevention of immune-related disorders, as represented by multiple sclerosis, has received considerable attention. The outbreak of the COVID-19 epidemic also prompted enthusiasm for research in this area. However, despite many hopeful and encouraging findings, there is no established consensus. The integral picture of the role of vitamin D in immunomodulation deserves and needs further study.

## Data availability statement

The original contributions presented in this study are included in the article/supplementary material, further inquiries can be directed to the corresponding author.

## Author contributions

WH proposed the conception of this study and critically revised the manuscript. XL and YD conducted the data collection and analysis. XL wrote the draft of the manuscript. All authors contributed to the article and approved the submitted version.
